# Development and validation of a multi-dimensional measure of intellectual humility

**DOI:** 10.1371/journal.pone.0182950

**Published:** 2017-08-16

**Authors:** Mark Alfano, Kathryn Iurino, Paul Stey, Brian Robinson, Markus Christen, Feng Yu, Daniel Lapsley

**Affiliations:** 1 Ethics & Philosophy of Technology, Delft University of Technology, Delft, Netherlands; 2 Psychology Department, University of Oregon, Eugene, Oregon, United States of America; 3 Center for Biomedical Informatics, Brown University, Providence, Rhode Island, United States of America; 4 Philosophy Department, Texas A & M University- Kingsville, Kingsville, Texas, United States of America; 5 Institute for Biomedical Ethics and History of Medicine, University of Zurich, Zurich, Switzerland; 6 Institute of Social Psychology, School of Humanities and Social Sciences, Xian Jiaotong University, Xian Shi, China; 7 Psychology Department, University of Notre Dame, South Bend, Indiana, United States of America; Georgetown University Medical Center, UNITED STATES

## Abstract

This paper presents five studies on the development and validation of a scale of intellectual humility. This scale captures cognitive, affective, behavioral, and motivational components of the construct that have been identified by various philosophers in their conceptual analyses of intellectual humility. We find that intellectual humility has four core dimensions: Open-mindedness (versus Arrogance), Intellectual Modesty (versus Vanity), Corrigibility (versus Fragility), and Engagement (versus Boredom). These dimensions display adequate self-informant agreement, and adequate convergent, divergent, and discriminant validity. In particular, Open-mindedness adds predictive power beyond the Big Six for an objective behavioral measure of intellectual humility, and Intellectual Modesty is uniquely related to Narcissism. We find that a similar factor structure emerges in Germanophone participants, giving initial evidence for the model’s cross-cultural generalizability.

## Introduction

There are well-documented scales for humility as such Ashton and Lee [1], openness to experience [1], and need for cognition Cacioppo and Petty [2]. Since intellectual humility is conceptually distinct from these constructs, it would be valuable to have an intellectual humility scale as well. In this paper, we make a contribution to the burgeoning interdisciplinary field of character psychology by building and validating a psychological scale of intellectual humility. In doing so, we follow the best practices in scale construction which have been laid out by Leonard Simms [3] as a three-phase process:

1. the substantive validity phase,
2. the structural validity phase, and
3. the external validity phase.

In phase one, experimenters conduct a thorough literature review of the construct to be measured and related constructs. Based on this review, they conduct expert-review studies and develop an initial item pool. In phase two, the experimenters develop an item selection strategy, collect responses from appropriate samples, evaluate the items psychometrically, create provisional scales, and modify and add items to address problems. Finally, in phase three, the experimenters conduct studies to evaluate convergent, divergent, discriminant, and criterion-related validity, finalize their scale, and report it.

### Substantive validity: Expert-review

For phase one, we collected conceptual definitions of the construct. No consensus emerges within philosophy or psychology on a precise definition of intellectual humility; primary contenders include Hazlett [4], Roberts and Wood [5–6], Samuelson and Church [7], Whitcomb et al. [8], Samuelson et al. [9], and Christen et al. [10]. Hazlett [4] thinks that intellectual humility is the “disposition not to adopt epistemically improper higher order epistemic attitudes, and to adopt […] epistemically proper higher order epistemic attitudes.” This conception of intellectual humility is most pertinent in the realm of disagreement. Hazlett’s idea is that, when you disagree with an epistemic peer (someone who is roughly as well-positioned as you to know the truth about the disagreed-upon proposition), you manifest intellectual humility neither by suspending judgment nor by revising your first-order belief, but by giving up the meta-belief that your first-order belief is correct. For example, suppose that, in January 2016, you predict that Donald Trump will become president of the United States, whereas I predict that it will be Hilary Clinton. We are both political news junkies, pay attention to data-driven electoral predictors, and monitor political betting markets. More generally, we’re both intelligent, reasonable people with no ulterior motives in this case. According to Hazlett, I can respond to our disagreement in an intellectually humble way even if I do not revise my prediction. Instead, he contends, all I need to do is think something like, “Clinton is going to be the president, but my position isn’t the only reasonable one.”

Roberts and Wood advance a similar view, holding that intellectual humility is “an unusually low dispositional concern for the kind of status that accrues to persons who are viewed by their intellectual communities as intellectually talented, accomplished, and skilled” [5]. They have since updated their definition to say that intellectual humility is “a striking or unusual unconcern for social importance, and thus a kind of emotional *insensitivity* to the issues of status” [6]. Both versions of this definition emphasize the social nature of intellectual humility. Roberts and Wood put more weight on the intellectually humble person’s concerns and emotions, while Hazlett focuses more on her doxastic states.

Samuelson and Church [7] characterize intellectual humility in the dual-process language popular in contemporary psychology. They claim that intellectual humility is “mostly found in the conscious exercise of Type 2 thinking and can come about […] through the proper collaboration of Type 1 and Type 2 processes […] or through the conscious practice of applying Type 2 thinking” [7]. Thus, Samuelson and Church think that intellectual humility can be implemented as a motivating trait, but they are more inclined to construe it in the dual-process framework, where it harmonizes automatic processes (heuristics, affective intuitions, etc.) with slow, controlled, effortful, attentive thought and deliberation. On this view, someone who tends to jump to conclusions based on the intuitive deliverances of System 1 fails to be intellectually humble, especially if he is not open to revising his beliefs in the face of new evidence. By contrast, someone who forces himself to slow down and think carefully in situations where intuitive responses are liable to mislead would be a paragon of intellectual humility. Whereas Hazlett and Roberts and Wood focus primarily on the context of disagreement and intellectual hierarchies, Samuelson and Church seem to think that intellectual humility operates more widely, fine-tuning the cognitive agent’s credences and inquiries whether or not other people disagree.

Whitcomb et al. [8] propose a sophisticated conception of intellectual humility as appropriately attending to and owning one’s cognitive limitations. Such attentiveness can be conscious, but it is grounded in an implicit sensitivity to one’s own dispositions. Attending to one’s limitations is in turn meant to lead to intellectually humble cognitive, behavioral, motivational, and affective responses. This multi-track trait leads the intellectually humble person to revise her beliefs in light of her recognition of her limitations, to try to overcome or quarantine the bad effects of her limitations, to desire to embody fewer and less severe limitations, and to display fitting emotions towards her limitations.

Samuelson et al. [9] give a descriptive rather than a normative account of intellectual humility. They find that it decomposes into three clusters or dimensions: openness to new ideas and knowledge, agreeableness and honesty in the context of (potential) disagreement, and modest unpretentiousness and reluctance to brag. Finally, like Samuelson et al. [9], Christen, Robinson, and Alfano [10] give a descriptive rather than a normative account of intellectual humility. They think that intellectual humility can be understood as a multifaceted disposition that opposes other dispositions. Rather than consulting their own intuitions about what the facets of intellectual humility and its opposing vices are, however, they employed a novel thesaurus-based psycholexical analysis, which suggests that intellectual humility has three positive facets (the sensible self, the discreet self, and the inquisitive self) and three opposing vices (the underrated other, the underrated self, and the overrated self). The sensible self is characterized by comprehension, responsiveness, and mindfulness—all ways of demonstrating openness to new ideas and information. The inquisitive self is characterized by curiosity, exploration, and learning—all ways of seeking new ideas and information. The discreet self is characterized by demureness and unpretentiousness—ways of relating to other people, especially those one might disagree with. This account is perhaps the most capacious, including self- and other-oriented facets, as well as dispositions to respond in characteristic ways to new ideas, to seek out new information, and to be mindful of others’ feelings and reactions in intellectual engagements.

### Substantive validity: Existing scales

In the previous section, we showed that experts on intellectual humility tend to see it as a multi-faceted disposition that directs cognition, emotion, and behavior both in social contexts and in solitary inquiry. To determine whether a new scale of intellectual humility is warranted, we now survey the literature. When we began this project, there were no published scales of intellectual humility. There are currently three such scales:

1. The General Intellectual Humility Scale developed by Leary et al. [11], a 6-item uni-factorial measure of intellectual humility,
2. The Comprehensive Intellectual Humility Scale by Krumrei-Mancuso & Rouse [12], a four-factor measure of aspects of intellectual humility, and
3. A bi-factorial measure of intellectual humility by McElroy et al. [13].

Naturally, we were not aware of these scales when we developed our own, so we do not wish to claim that ours is decisively better. Further testing is likely needed to directly compare these scales. Instead, we expect that each scale may have value in different contexts, and that future work may distinguish among them or result in a hybrid.

That said, we should mention a few key differences between these scales and the one presented here, which point to potential advantages of our scale. First, the Leary et al. [11] scale is uni-factorial, while the McElroy et al. [13] scale is bi-factorial. By contrast, the scale we present here has four factors, like the one developed by Krumrei-Mancuso & Rouse [12]. This is important because our expert-review in the previous section suggests that intellectual humility is conceived of as multi-dimensional. A one- or two-dimensional scale of intellectual humility cannot capture as much of the breadth of the construct as a four-dimensional scale. In addition, a multi-dimensional scale enables us to see how different aspects of intellectual humility may relate differently to constructs of interest, thus potentially giving us a more complete theoretical understanding of intellectual humility. Second, from a psychometric point of view, none of the existing scales of intellectual humility delves into the fine-grained properties of the performance of specific scale items across the full range of the latent trait of intellectual humility. In this paper (Study 2), we conduct an item-response theoretic analysis of our scale items. None of the other scales have been analyzed in this way, so our scale provides more analytical texture than any of the existing scales can boast. Third, the McElroy et al. [13] scale exists only in an informant-report form, whereas the other two existing scales exist in self-report form. The scale we develop here exists in both forms and is therefore the first to unify self-perception and informant-perception of intellectual humility. Using both forms is important because the expert-review in the previous section suggests that intellectual humility has both social and solitary aspects. It may be especially pertinent in the context of social disagreement, but intellectual humility is also relevant when one is on one’s own. Fourth, convergent, divergent, and discriminant validity have not been decisively demonstrated for any extant measure of intellectual humility, though there are some positive indications for Leary et al. [11] and Krumrei-Mancuso and Rouse [12]. We subject our scale to much more extensive tests of these aspects of validity in Study 4. Fifth, extant work on intellectual humility has used only English-speaking samples. This may be unsurprising, but we believe that it is important to tune psychological measures not only to English-speakers but also to the rest of the human population. Therefore, in this paper (Study 5), we make a first foray by translating our scale into German and performing both exploratory and confirmatory factor analyses with a German-speaking population. Sixth, because the paper by Leary et al. [11] is still under review, the details of its methodology are only available second-hand through other papers that have cited it. This makes it difficult to assess the quality and reproducibility of their scale. In this paper, we report multiple studies with large samples that demonstrate reproducibility in both the same population and different populations. Seventh, and in a related vein, our scale was developed using samples roughly an order of magnitude larger than those used in the scale presented by Krumrei-Mancuso and Rouse [12]. In particular, when testing convergent, divergent, and discriminant validity (study 5), we employed N = 980 while Krumrei-Mancuso and Rouse (study 4) employed just N = 179. This suggests that the reliability and reproducibility of our scale may be greater. Ninth, the McElroy et al. [13] scale was developed in a very narrowly defined population: religious leadership. Positions of power in religious organizations surely do demand intellectual humility, but so do many other positions, whether empowered or not. Finally, in contrast with the closely-held data and code associated with the other scales, we have made all of our (anonymized) data and code open and available for other researchers (https://github.com/paulstey/IntellectualHumility), thus contributing to open and reproducible science. For these reasons, we suggest that our scale may prove more attractive to many researchers and empirically-informed policy-makers than the three existing scales canvassed above.

In sum, we have endeavored to develop a model of intellectual humility that provides broad conceptual coverage. In addition, we have aimed to develop a measure with good psychometric characteristics, validity, and interpretability, in order to supplement and advance the theoretical views of intellectual humility described above. In the remainder of this paper, we describe three studies that establish the structural validity of the scale, followed by two studies related to its external validity. We conclude with a general discussion of our research and an exploration of the prospects for future research.

## Structural validity: Study 1: Factor structure of intellectual humility

### Methods

#### Ethics statement

This research was approved by the IRBs of the University of Notre Dame and Grand Valley State University (Study 1), University of Oregon (Studies 2, 3, and 4), and University of Zurich (Study 5). In all studies, participants provided written consent after reading a brief description of they study by ticking a box and clicking on a button. If consent was not provided, participants were not allowed to proceed with the study.

#### Participants and procedure

Participants were college students at a large Midwestern American public university. This was a sample of convenience from which we generalized in later studies. We sent an invitation email to a random sample of 5000 students, of whom 442 responded. The average age of participants was 20.9; about 90% of participants were between the ages 18–22. 30.5% of the sample was male. 85.5% of the sample was White/Caucasian, 4.5% was African-American/Black, 2.9% was Asian, and 1% was Hispanic. Average GPA of the sample was 3.29, the average SAT score was 1346.67, and the average ACT score was 25.44.

#### Measure

The original item pool consisted of 52 items (see S1 Table). Items were informed by a thorough consideration of the defining aspects of intellectual humility, as well as some of its more penumbral elements, based on a thorough literature review of psychological and philosophical research on humility in general—Tangney [14–15]; Lee & Ashton [16]; Ashton & Lee [1]; Davis et al. [17]—and intellectual humility specifically—Roberts & Wood [5–6]; Hazlett [4]; Samuelson & Church [7]; Zagzebski [18]; Whitcomb et al. [8]; Christen, Robinson, & Alfano [10]—as summarized in the introduction. We aimed to include items that answered to at least one definition of intellectual humility, casting a wide net so as to include items even if one or more of the above definitions would rule them out. Other items were adapted from items in existing scales that intuitively seem to be related to intellectual humility as we characterized it above. In particular, we adapted several items from the HEXACO personality inventory [1], including most subscales of the honesty/humility dimension, as well as the dependence subscale of emotionality, the diligence subscale of conscientiousness, and the inquisitiveness and unconventionality subscales of openness.

Throughout, we were mindful that intellectual humility is an unusual and potentially tricky disposition to measure because it seems to involve a paradox of self-attribution. If you say or even think that you are humble, it’s unlikely that you are humble. If you are humble, it’s unlikely that you’ll think so, and even more unlikely that you’ll say so. We tried to avoid language that would trigger this paradox.

A balance of reverse- and forward-keyed items was included to tap dispositions contrary to intellectual humility and to account for participant acquiescence. Participants were asked to rate on a 7-point scale their level of agreement with the items, with 1 anchored as “strongly disagree” and 7 anchored as “strongly agree.”

#### Results

To determine the factor structure, we used M*plus* Version 7, developed by Muthén & Muthén, [19] to conduct an exploratory factor analysis (EFA) using ML with promax rotation. A parallel analysis suggested 7 factors should be extracted by strict cut-off criteria, but the 8-factor solution was more interpretable. Upon inspection of the content of each factor, we chose names for the eight factors as shown in Table 1. As T. S. Eliot emphasized, the naming of cats (and factors) is a difficult matter. The names we chose were arrived at by consensus among the researchers after extensive discussion. We are more pleased with some of them (engagement, uniqueness, curiosity, Machiavellianism) than others (open-mindedness, modesty). Nevertheless, we think that the labels provided in Table 1 are adequate to the job. In particular, reasonable people might suggest that the factor we label ‘open-mindedness’ should be labeled ‘modesty’. However, because the items associated with this factor tend to focus on cases in which one either is ignorant when someone else is knowledgeable or in which one disagrees with another person but has the opportunity to take their view seriously, we think that ‘open-mindedness’ is the most appropriate label.

**Table 1:** Names and qualitative descriptions of the 8 factors suggested by the exploratory factor analysis of the results of study 1. In bold are the factors determined to be central to intellectual humility.

Name	This dimension involves…
**Open-mindedness** (negative pole: Intellectual Arrogance)	Behavior and attitudes that reflect an acknowledgment of the limitations of one’s knowledge, especially relative to others (rather than arrogance about one’s intellectual capabilities and knowledge), and a desire to gain knowledge irrespective of status.
**Intellectual Modesty** (negative pole: Intellectual Vanity	Low concern for how one’s intellect is perceived, and for one’s intellectual reputation.
**Engagement** (negative pole: Boredom)	Motivation to investigate things one doesn’t understand, particularly in response to encountering ideas different from one’s own.
**Corrigibility** (negative pole: Intellectual Fragility)	Resilience in emotional response when confronted with challenges to one’s knowledge or intellectual abilities.
Intellectual Uniqueness	Feeling special when one has knowledge.
Curiosity	High levels of tenacity applied to mastering new concepts.
Intellectual Machiavellianism	Manipulating others to get information or knowledge.
Intellectual Kleptomania	Taking credit for ideas that aren’t one’s own.

In the 7-factor solution, six of the factors were identical to the factors in the 8-factor solution. The main drawback of the 7-factor solution was that in it, the Curiosity items were lumped with some of the Open-mindedness items. Though the items loading on the Open-mindedness/Curiosity factor in the 7-factor solution have a common emphasis on learning, the Open-mindedness factor in the 8-factor solution reflects an absence of arrogance, and a prioritization of learning regardless of one’s intellectual status. This insensitivity to status and lack of arrogance more specifically embody the Open-mindedness construct than the more general Open-mindedness/Curiosity factor. Curiosity is likely to be important to Open-mindedness to the extent that it motivates one to understand new ideas. However, it is plausible that one could be intellectually determined while also being intellectually arrogant. Therefore, for conceptual clarity, we opted for the 8-factor solution that treated these as distinct.

Items that had a loading greater than .30 in magnitude on their primary factor were retained (see S1 Table for items that met this criterion). Though ideally each item would also have low cross-loadings (< .10) on all factors other than the primary factor, only four items satisfied this criterion. However, compared to solutions with fewer factors, the average proportion of cross-loadings >.10 for each item was lowest in the 8-factor solution.

### Discussion

The 7- and 8-factor solutions were quite similar. Some of the factors, however, seemed to be peripherally related to intellectual humility. For instance, Intellectual Machiavellianism could be seen as just a specific kind of Machiavellianism, having little to do with how one is perceived, peer- and non-peer-disagreement, emotional reactions to ignorance and learning, and intellectual hierarchies. Intellectual kleptomania centered on the theft of ideas—a topic dear to university professors, lawyers, and hackers, but perhaps less interesting to the general populace. Intellectual Uniqueness was also problematic because only three items loaded on it, and all of them used the slightly odd phrase “feel special.” This led us to worry that the Intellectual Uniqueness items correlated with one another not because they tapped different parts of the same construct but simply because they were nearly synonymous. Finally, though in the seven-factor solution Curiosity tended to be lumped with Open-mindedness, based on the philosophical frameworks canvassed above, it seemed clear that Curiosity, while related to intellectual humility, is conceptually distinct from it. In particular, curiosity involves the inquisitive seeking-out of new evidence and the asking of questions, whereas intellectual humility has more to do with confronting existing intellectual problems and disagreements.

This first study used a sample of convenience of university students. In order to establish the model’s generalizability to the adult USA population, we next conducted a second study with non-student participants, the results of which were subjected to confirmatory factor analysis (CFA) and item response theoretic analysis [20]. With a separate sample of participants, we sought to confirm the 8-factor structure found in Study 1, and if confirmed, further develop the four subscales most central to intellectual humility.

## Structural Validity: Study 2: Replication of factor structure of intellectual humility

### Methods

#### Participants and procedure

Participants (*N* = 465; *M*_age_ = 33.29, 240 female) were recruited and compensated using Amazon.com’s Mechanical Turk platform. The Mechanical Turk population sampled in this study was more diverse than the university student population used in our first study in terms of age, educational attainment, and race/ethnicity. Ages ranged from 18–82; median education completed was an Associates degree; 41.9% had a Bachelors or higher level of education. Seventy-five percent of participants were White/Caucasian, 8.6% were African-American or Black, 7.7% were Asian, 5.8% were Hispanic, 0.4% were Pacific Islander, and 1.9% were Multiracial. These descriptive statistics are fairly similar to the population of the United States as a whole, where 29.3% of the adult population has a Bachelors Degree or equivalent, and where 77.1% of the population is White/Caucasian, 13.3% is African-American or Black, 5.6% is Asian, 17.6% is Hispanic, 0.2% is Pacific Islander, and 2.6% is Multiracial (according to the estimates based on the 2015 vintage of http://www.census.gov). It appears that highly-educated people are somewhat over-represented in our sample, while Blacks and Hispanics are somewhat under-represented.

#### Measure

The measure was identical to the one used in study 1. Participants responded to 52 items presented in random order.

#### Analyses

To determine whether the 8-factor structure replicated in a second sample of non-college students, we conducted a CFA *in MPlus* using Maximum Likelihood estimation with robust standard errors. Exploratory data analysis revealed the assumption of multivariate normality had been violated (Mardia’s coefficient = 69.82). In the CFA we included all items that loaded above .30 on their factors in study 1, and examined the fit according to several fit indices that indicate the degree of misspecification in the model: the comparative fit index (CFI), the root mean square error of approximation (RMSEA), and the standardized root mean square residual (SRMR). CFI ranges from 0 to 1, with values closer to 1 indicating better fit, and reflects the proportion of improvement in fit relative to the null (independence) model. RMSEA and SRMR are measures of absolute fit, that is, how well on average the correlation matrix has been reproduced by the model. According to Hu and Bentler [21], CFI should ideally be greater than .95, RMSEA should be less than .06, and SRMR should be less than .08. By the most conservative standard, a model should meet all these benchmarks. A more lenient standard allows for lower values (CFI .90 to 95, RMSEA .06 to .08, SRMR .08 to .10) as indicative of a marginally fitting model.

### Results

#### Confirmatory factor analysis

The CFA indicated the 8-factor model had good fit according to RMSEA and SRMR, but was not adequate according to CFI benchmark (*χ*^*2*^(1052) = 2400.97, CFI = .83, RMSEA = .05, SRMR = .08).

Therefore, we looked for sources of misspecification in the model. Inspection of the modification indices in M*plus* indicated that a main source of misfit were a few items’ tendency to cross-load on multiple other factors besides their primary factor. To maintain our measure’s broad coverage of the construct while maximizing interpretability of each construct, we sought to separate out these factors from one another as much possible. Therefore, we first sought to remove items based on their tendency to cross-load on multiple factors (in particular, items 14 and 16 from the modesty factor, and 39 and 41 from the Open-mindedness factor were clear candidates for removal based on this criteria). Dropping these items resulted in a model with better fit according to all indices, *χ*^*2*^(874) = 1742.62, CFI = .87, RMSEA = .05, SRMR = .06.

As described previously in study 1, the content of four of the factors (Intellectual Machiavellianism, Intellectual Kleptomania, Intellectual Uniqueness, and Curiosity), though perhaps related to intellectual humility, was more peripheral to the target construct of intellectual humility. That these four factors are more peripheral is also supported by the fact that the pairwise correlations between core factors (Open-mindedness, Intellectual Modesty, Engagement, and Corrigibility) tended to be higher than those between any given peripheral factor and a core factor (e.g., between Open-Mindedness and Intellectual Kleptomania). Therefore, the rest of this paper focuses on the refinement and validation of these four subscales.

We next examined each of the four core factors separately, with the goal of creating four unidimensional subscales. We evaluated the performance of items on each scale based on the following criteria: 1. correlation with the scale-total, 2. variance of item’s correlations with other items in the scale, as an index of unidimensionality, 3. contribution to the breadth of the scale’s conceptual coverage, 4. contribution to the balance of forward- and reverse-keyed items in the scale, and 5. contribution to diversity in difficulty level of items, as indicated by the item means. Weighing these considerations, we further removed items 17, 23, 28, 33, 36, and 52 from the Open-mindedness factor (leaving items 27, 34, 35, 45, 50, 51) and items 9 and 12 from the Intellectual Modesty factor (leaving items 8, 10, 11, 13, 15, and 32).

In addition, the former Open-mindedness item 39, *“I appreciate being corrected when I make a mistake*,*”* was added to the Corrigibility scale to see how it would perform as part of that scale. As described previously, the CFA suggested this item should load on the Corrigibility factor, and this item was well-grouped with the other items in the Corrigibility scale in terms of content, as they are all about how one responds emotionally to being criticized in some intellectual domain. Additionally, unlike the other items on the scale, this item is one of only two non-reverse-scored items in the Corrigibility scale, making it an even more potentially valuable component of that scale.

For the Corrigibility and Engagement subscales, the items were generally comparable in their strengths on the selection criteria, so no items were removed from those scales. Although a few items in these scales still had low factor loadings, they were kept because they had other merits, such as increasing the scale’s breadth of representation of the construct in terms of content, keying, or range of difficulty in endorsement (as indicated by the item means).

After trimming all these items, and adding item 39 to the Corrigibility subscale, the four-factor solution had better fit, *χ*^*2*^(224) = 513.30, CFI = .90, RMSEA = .05, SRMR = .06. These fit indices meet Hu and Bentler’s [21] standards for RMSEA and SRMR, and more lenient thresholds for CFI. (See Table 2 for the final four-factor solution from Study 2, and standardized factor loadings).

**Table 2:** The final 4-Factor solution from Study 2 after CFA.

	OPM	MOD	COR	ENG
27. I think that paying attention to people who disagree with me is a waste of time.	-.64			
34. I feel no shame learning from someone who knows more than me.	.67			
35. If I do not know much about some topic, I don't mind being taught about it, even if I know about other topics.	.74			
45. Even when I have high status, I don't mind learning from others who have lower status.	.71			
50. Only wimps admit that they've made mistakes.	-.67			
51. I don't take people seriously if they're very different from me.	-.74			
8. Being smarter than other people is not especially important to me.		.69		
10. I would like to be seen explaining ideas that no one else understands.		-.58		
11. I get a lot of pleasure from knowing more than other people.		-.73		
13. I wouldn't want people to treat me as though I were intellectually superior to them.		.47		
15. I want people to know that I am an unusually intelligent person.		-.69		
32. I like to be the smartest person in the room.		-.78		
37. I find it annoying to be told that I've made an intellectual mistake.			-.70	
38. If someone points out an intellectual mistake that I've made, I tend to get angry.			-.82	
39. I appreciate being corrected when I make a mistake.			.67	
40 When someone corrects a mistake that I've made, I do not feel embarrassed.			.55	
43. When I realize that someone knows more than me, I feel frustrated and humiliated.			-.75	
18. I rarely discuss things that I wish I understood better with other people.				-.57
24. I enjoy reading about the ideas of different cultures.				.48
25. I would be very bored by a book about ideas I disagreed with.				-.53
26. I’ve never really enjoyed figuring out why people disagree with me.				-.57
29. I find it boring to discuss things I don't already understand.				-.64
31. A disagreement is like a war.				-.53

Note. OPM = Open-mindedness, MOD = Intellectual Modesty, COR = Corrigibility, and ENG = Engagement. Factor loading estimates are STYDX. N = 465.

#### Item response theory analysis

In order to investigate the psychometrics of our measure, we supplemented the confirmatory factor analysis with analyses from the perspective of item response theory (IRT). IRT is a model-based framework used for investigating item and test properties; it assumes a latent trait or ability that is a function of both the participants’ responses, and the properties of the items [22]. Thus, IRT allows us to estimate both an individual’s trait level and the relevant item parameters.

The goal of this further examination was two-fold. First, we aimed to identify the characteristics of individual items; second, we wanted to estimate the overall reliability of the measure in a manner distinct from the classical testing theory approach. In order to investigate the individual properties of items, we used a graded response model [23–24] implemented in the ltm package [25] in the R statistical language [26].

Of particular interest were item slopes and threshold parameters. Item slopes describe an item’s ability to differentiate between participants having levels of the latent trait above or below the item’s location [27]. Item slopes are frequently referred to as discrimination parameters. Such parameters can be considered cut points on the latent trait’s continuum where a participant with that level of the latent trait is equally likely to select the response category *j* rather than category *j* + 1.

Table 3 shows the parameter estimates from the fitted response model of the Open-mindedness items. Several features are important to note. First, the threshold parameter estimates (denoted by *b*) are distributed towards the negative end of the latent continuum; we revisit this below. Second, the standard error estimates are all rather small even at the extremes of the response scale. This suggests participants made use of the entirety of the response categories, a desirable feature. Finally, we note that the slope parameters are quite similar across the various items. This is encouraging in that it supports the use of simple un-weighted sum scoring to compute the scale [28].

**Table 3:** Intellectual humility–Open-mindedness item parameter estimates.

Item	*b*_1_	*b*_2_	*b*_3_	*b*_4_	*b*_5_	*b*_6_	*a*
27	–4.14 (0.53)	–2.81 (0.53)	–1.87 (0.49)	–1.57 (0.48)	–0.60 (0.46)	0.90 (0.47)	1.61 (0.15)
34	–3.09 (0.29)	–2.60 (0.37)	–2.27 (0.36)	–1.86 (0.34)	–1.15 (0.31)	0.25 (0.27)	2.48 (0.22)
51	–3.17 (0.31)	–2.30 (0.35)	–1.76 (0.32)	–1.35 (0.31)	–0.71 (0.28)	0.75 (0.28)	2.08 (0.18)
45	–3.10 (0.30)	–2.78 (0.38)	–2.21 (0.35)	–1.61 (0.32)	–0.68 (0.29)	0.69 (0.28)	2.42 (0.21)
50	–3.30 (0.36)	–2.62 (0.42)	–2.26 (0.41)	–1.88 (0.39)	–1.39 (0.37)	–0.11 (0.33)	2.41 (0.23)
35	–3.41 (0.42)	–2.67 (0.49)	–2.28 (0.47)	–1.66 (0.45)	–0.93 (0.43)	0.44 (0.40)	2.70 (0.24)

Note: *b* indicates a threshold parameter, *a* indicates slope, *SE* estimates appear in parentheses.

We also examined the item properties of the other factors: Intellectual Modesty (Table 4), Corrigibility (Table 5), and Engagement (Table 6). For Intellectual Modesty, we observe that the threshold parameters suggest a more balanced distribution across the latent continuum than was observed with the Open-mindedness items. We also observe that the *SE* estimates are generally small; there are exceptions, however. For the final threshold parameter, *b*_6_, several of the items have *SE* estimates that are quite large. This indicates imprecision in the estimation of that parameter, and could be a function of the small number of items in the scale or the sample size. For Corrigibility and Engagement, we again see that the item threshold estimates are well distributed across the latent continuum.

**Table 4:** Intellectual humility–Intellectual modesty item parameter estimates.

Item	*b*_1_	*b*_2_	*b*_3_	*b*_4_	*b*_5_	*b*_6_	*a*
8	-2.23 (0.17)	-1.17 (0.19)	-0.37 (0.16)	0.15 (0.15)	0.66 (0.23)	1.97 (2.48)	2.01 (0.17)
10	-2.37 (0.21)	-1.09 (0.19)	-0.05 (0.14)	0.96 (0.25)	1.53 (0.87)	2.92 (6.05)	1.42 (0.13)
11	-1.96 (0.14)	-0.87 (0.17)	0.08 (0.12)	0.74 (0.23)	1.18 (0.91)	2.23 (9.06)	2.25 (0.19)
13	-3.54 (0.38)	-2.29 (0.35)	-1.37 (0.29)	-0.53 (0.24)	0.46 (0.25)	1.94 (0.72)	1.12 (0.12)
15	-2.49 (0.2)	-1.4 (0.23)	-0.47 (0.18)	0.37 (0.17)	0.92 (0.37)	1.95 (2.75)	1.98 (0.17)
32	-1.88 (0.13)	-0.97 (0.17)	-0.22 (0.14)	0.44 (0.14)	0.94 (0.51)	1.82 (5.01)	2.54 (0.23)

Note: *b* indicates a threshold parameter, *a* indicates slope, *SE* estimates appear in parentheses.

**Table 5:** Intellectual humility–Corrigibility item parameter estimates.

Item	*b*_1_	*b*_2_	*b*_3_	*b*_4_	*b*_5_	*b*_6_	*a*
37	-2.26 (0.17)	-1.31 (0.2)	-0.44 (0.16)	-0.04 (0.15)	0.66 (0.21)	1.85 (2.11)	2.05 (0.17)
38	-2.73 (0.22)	-1.89 (0.32)	-1.07 (0.28)	-0.67 (0.26)	-0.09 (0.23)	1 (0.5)	2.88 (0.28)
39	-2.84 (0.24)	-1.89 (0.28)	-1.27 (0.25)	-0.7 (0.22)	0.35 (0.2)	1.73 (1.07)	1.84 (0.16)
40	-2.64 (0.24)	-1.37 (0.23)	-0.28 (0.17)	0.07 (0.18)	0.92 (0.28)	2.55 (2.95)	1.49 (0.14)
43	-2.98 (0.26)	-2.26 (0.34)	-1.12 (0.28)	-0.78 (0.26)	-0.22 (0.25)	1.09 (0.39)	2.21 (0.2)

Note: *b* indicates a threshold parameter, *a* indicates slope, *SE* estimates appear in parentheses.

**Table 6:** Intellectual humility–Engagement item parameter estimates.

Item	*b*_1_	*b*_2_	*b*_3_	*b*_4_	*b*_5_	*b*_6_	*a*
18	-3.06 (0.31)	-2.08 (0.32)	-1.14 (0.26)	-0.56 (0.23)	0.38 (0.22)	1.78 (0.94)	1.57 (0.16)
24	-4.31 (0.57)	-3.54 (0.58)	-2.41 (0.5)	-1.69 (0.46)	-0.52 (0.41)	1.08 (0.44)	1.24 (0.14)
25	-3.04 (0.31)	-1.87 (0.3)	-0.96 (0.24)	-0.32 (0.21)	0.48 (0.22)	1.86 (0.9)	1.39 (0.14)
26	-3.52 (0.39)	-2.11 (0.38)	-1.22 (0.34)	-0.58 (0.31)	0.35 (0.3)	1.74 (0.89)	1.57 (0.16)
29	-3.14 (0.34)	-2.13 (0.37)	-1.34 (0.32)	-0.93 (0.31)	-0.16 (0.28)	1.11 (0.43)	1.98 (0.2)
31	-4.31 (0.55)	-2.59 (0.47)	-1.55 (0.4)	-0.96 (0.37)	-0.12 (0.35)	1.44 (0.48)	1.09 (0.13)

Note: *b* indicates a threshold parameter, *a* indicates slope, *SE* estimates appear in parentheses.

**Fig 1:**
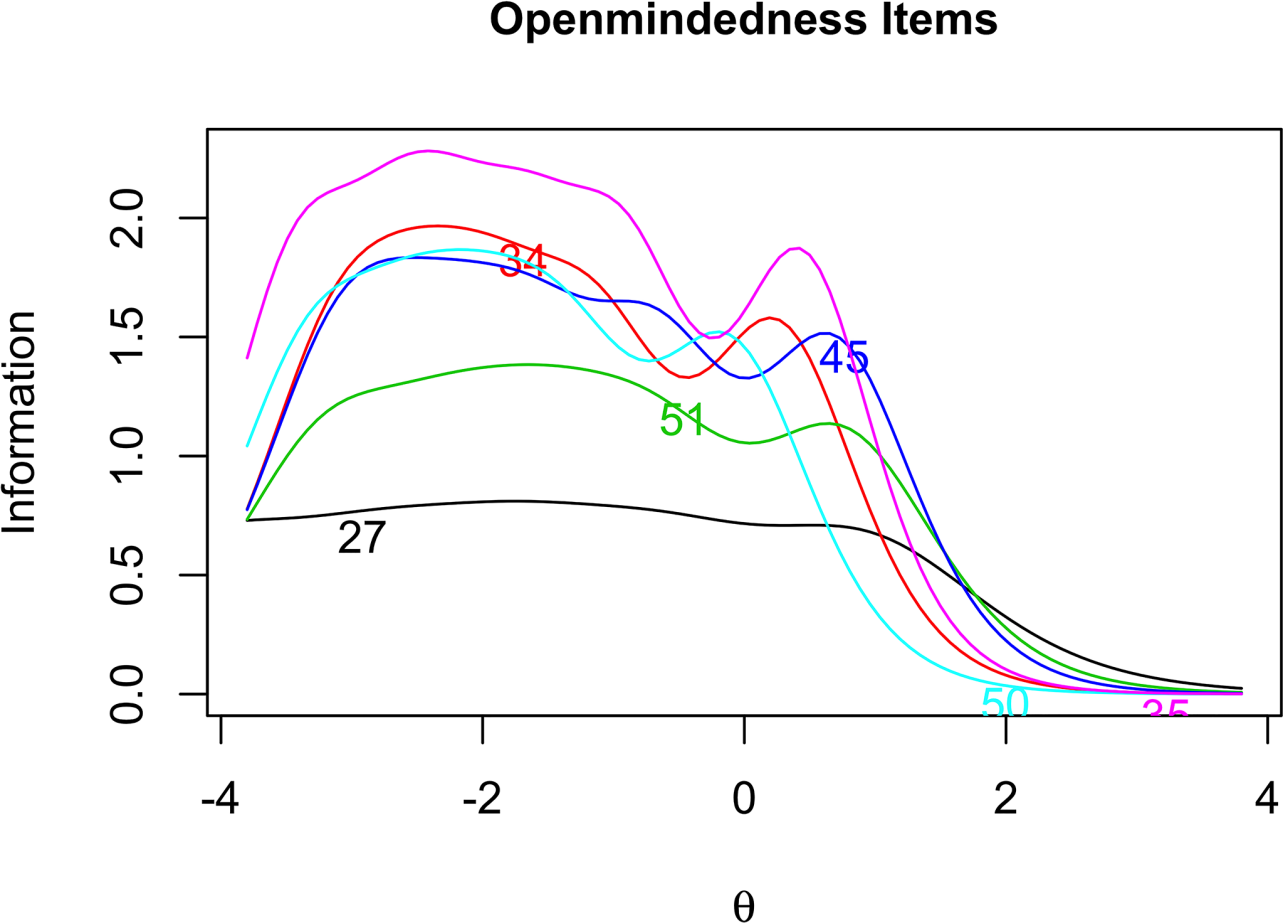
Open-mindedness item response analysis. Item information curves for Open-mindedness items.

**Fig 2:**
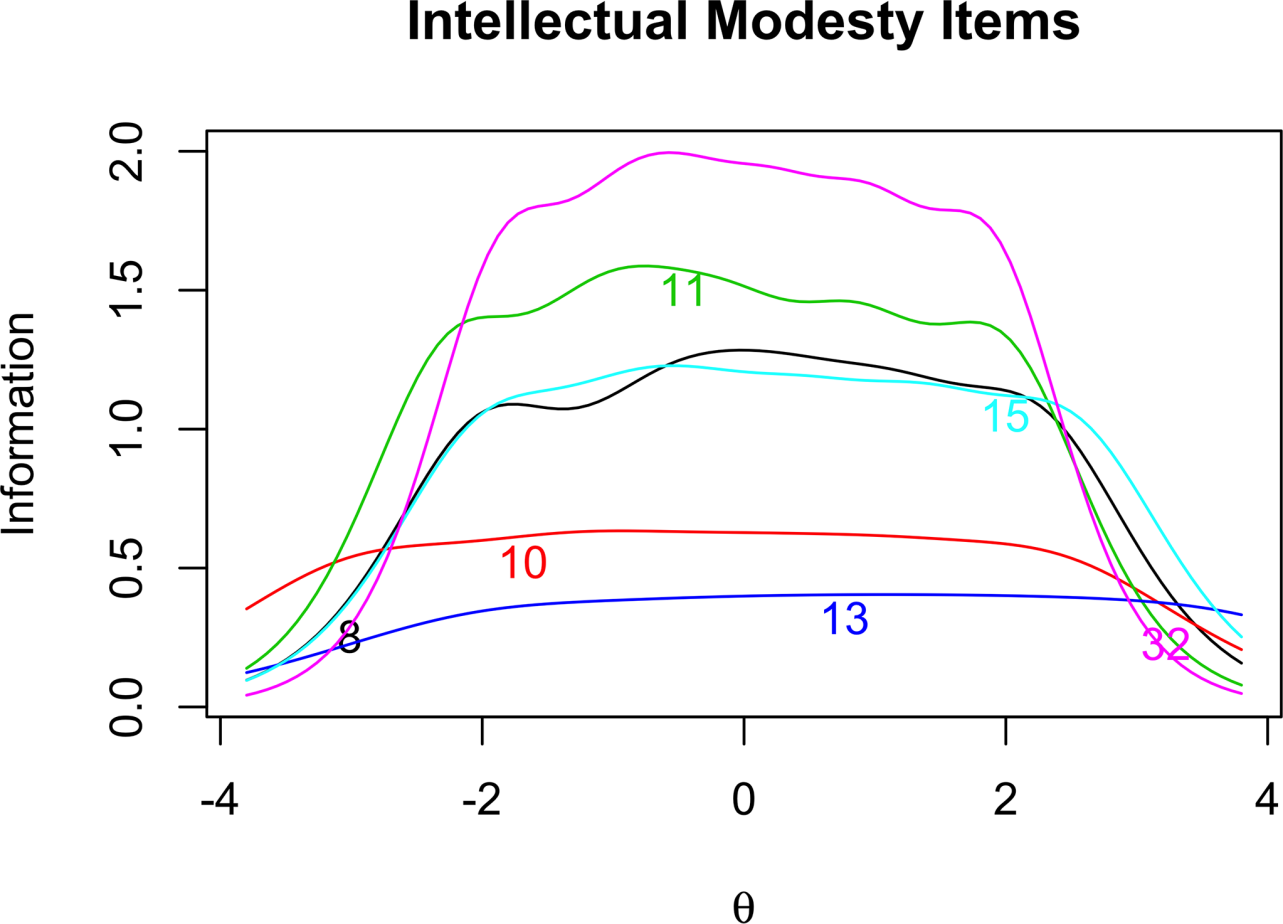
Intellectual modesty item response analysis. Item information curves for Intellectual Modesty items.

**Fig 3:**
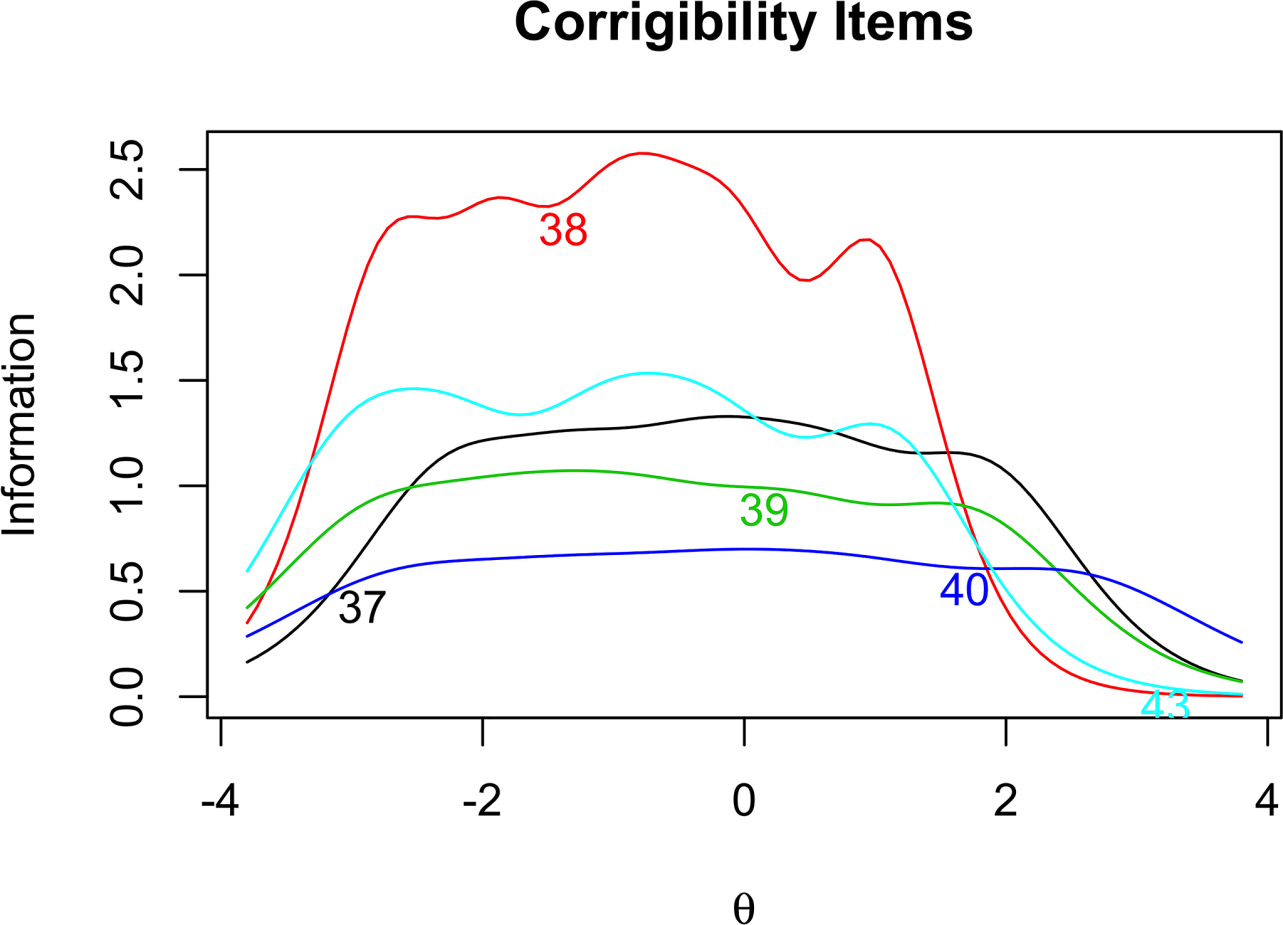
Corrigibility item response analysis. Item information curves for Corrigibility items.

**Fig 4:**
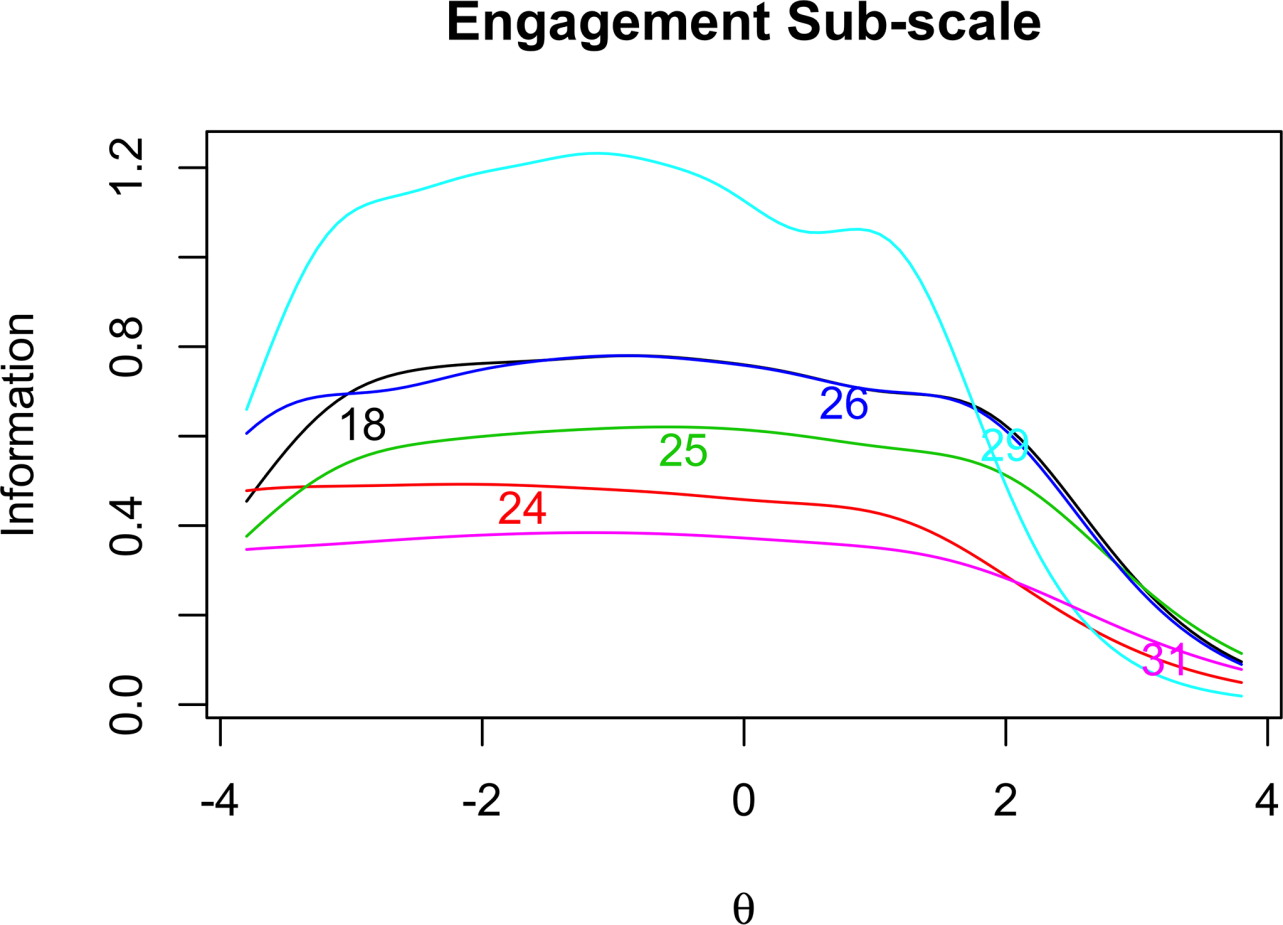
Engagement item response analysis. Item information functions for Engagement items.

In order to further investigate the properties of our items, we examine the item information curves for Open-mindedness (Fig 1), Intellectual Modesty (Fig 2), Corrigibility (Fig 3), and Engagement (Fig 4). Of particular importance in Fig 1 is the tendency for the Open-mindedness items to be particularly informative across the lower end of the latent continuum. We see that these items are most informative across the range of –4 to +1.5 on the latent trait continuum. This might give some reason for pause, as we would hope to see more even coverage across the latent continuum. Next, as Fig 2 shows, the Intellectual Modesty items have good coverage across the central portion of the latent continuum. This is encouraging and suggests that these items are generally well suited to measuring the construct across a broad range of participants. Two of our Modesty items, however, (Item 10, and particularly Item 13) are rather minimally informative. Regarding Fig 3, we note that Corrigibility items provide rather good coverage across the latent trait continuum, albeit with some partiality towards the positive end of the spectrum. In addition, we note that Item 38 seems to be particularly informative relative to the other Corrigibility items. Finally, the Engagement items were quite informative across the central and lower end of the latent trait continuum. Item 29 also emerges as particularly informative relative to the other items. Perhaps this is not surprising, given the wording of the item (i.e., “*I find it boring to discuss things I don’t already understand*.”). We would intuitively expect this to be very central to the underlying construct.

**Fig 5:**
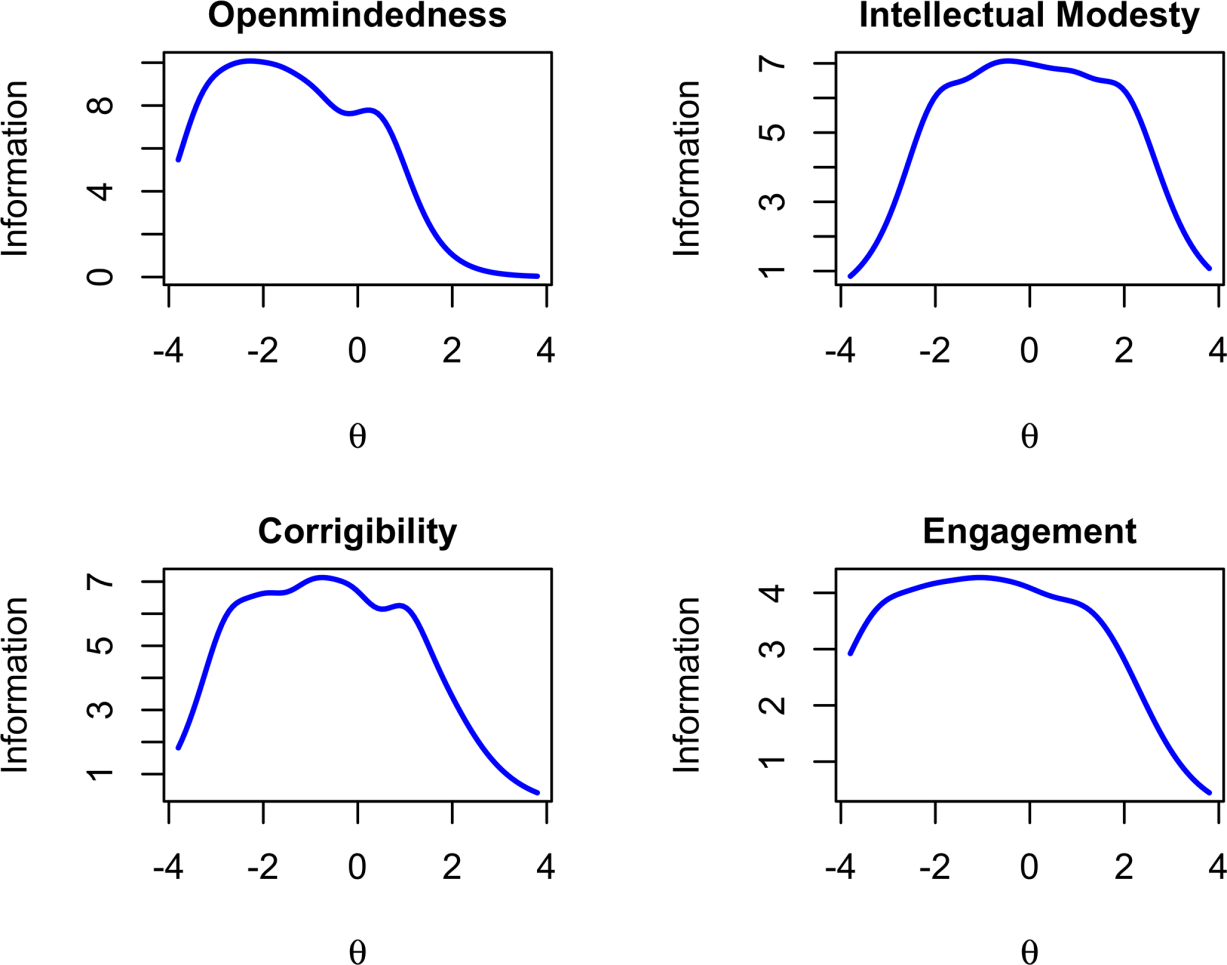
Intellectual humility item response analysis. Test information functions for subscales of the Intellectual Humility scale.

Lastly, we examine the test information functions for the 4 subscales discussed above (Fig 5). The subscales are especially informative in the central and lower parts of the latent continuum. We suspect this is related to ceiling and floor effects for certain items. Of the 4 subscales, the items measuring the modesty dimension seem most evenly distributed over the center of the latent continuum.

### Discussion

In light of the confirmatory factor analysis described above, we eliminated some items and factors to generate a four-factor scale of intellectual humility. This is a novel measure of an important construct, and the factor analysis suggests that our scale captures multiple dimensions of this complex construct. In addition, IRT indicates that the items tap different levels of the underlying factors, with especially good coverage at the centers and lower ends of the continua. As with any new measure, this scale has some drawbacks, including less than ideal coverage of some of the upper extremes of some of the subscales. To try to fill those gaps, we decided, for subsequent research, to add a few pilot items and tweak a few of the remaining items. Please see S1 Table for a description of modified (13*, 35*, 50*) and new items (53, 54, 55).

## Structural validity: Study 3: Informant report replication

Intellectual humility isn’t just a matter of what you think about yourself. Like any character trait, intellectual humility is a disposition to think, feel, and act. The characteristic manifestations of this disposition are often social (e.g., when and how someone responds to peer disagreement or intellectual criticism). So it would be surprising if someone who embodied intellectual humility were not seen as intellectually humble by those who know her well. For this reason, we next conducted an “informant” study of intellectual humility. Informant studies have become popular in personality psychology [29–33] in part because they lend external verification to self-report measures of dispositions. I might think that I’m the life of the party, but if everyone I know sees me as a wallflower, I’m probably wrong. Of course, intersubjective agreement isn’t infallible. I and my friends might all think that I’m uproariously funny despite my being a charmless bore. That said, informant reports shed some evidential light on people’s dispositions.

### Methods

#### Participants and procedure

Two groups of participants were recruited for this study. The first, self-report group of participants (*N* = 1185; *M*_age_ = 33.6, 56% female) were recruited and compensated using Amazon.com’s Mechanical Turk platform. This enabled us to perform an exact replication of the confirmatory factor analysis in Study 2. Ages ranged from 18–79, and median education completed was an associate’s degree; 43.5% had a Bachelors or higher level of education. 71.2% percent of participants were White/Caucasian, 13% were African-American or Black, 5.4% were Asian, 5.1% were Hispanic, .4% were Pacific Islander, and 1.8% were Multiracial.

The second, informant group of (*N* = 107; *M*_age_ = 36.12 years, 58% female) were recruited by emailing up to five informants per self-report participant. Participants had provided informants’ given names and email addresses. We then emailed all potential informants inviting them to tell us about the participant and offering as compensation a $5 online gift card to Target. Of the 1402 informants contacted, only *N* = 107 completed the survey, giving us informant ratings on 89 of our main participants (74 of our main participants had one informant, 14 had two informants, and one had five informants). Ages of the informants ranged from 16–65, 70.1% percent of informant participants were White/Caucasian, 6.5% were African-American or Black, 1.9% were Asian, 8.4% were Hispanic, .9% were Pacific Islander, and 3.7% were Multiracial.

#### Measure

Two distinct measures were used. For the self-report group, the measure was identical to the one used in studies 1 and 2 with the following exceptions. Participants responded to the original 52 items as well as the three new items (53, 54, and 55) and the modified items (13*, 35*, and 50*). Participants rated their agreement with these 58 items on a 7-point Likert-type scale.

For the informant group, the measure was identical to the one just described for the self-report group, but instead of asking participants to agree or disagree with statements about themselves (“I am….”), we asked them to agree with the same statements about the person who referred them to us (“s/he is….”). Informants were assured (truthfully) that their responses would not be shared with the primary participants. We also included four items meant to gauge how well the informants knew the participants.

### Results

#### Self-report analysis

Since we made a number of modifications to the original scale after the CFA analysis, we first sought to determine whether these modifications created a scale that performed well in an independent sample, or if development of the scale capitalized on chance characteristics of the sample in which the scale was derived. To evaluate this, we checked the overall model fit of the final four-factor version of the scale developed in Study 2. The final four-factor version of the scale had worse fit than it did in the sample where it was developed; however, by RMSEA and SRMR, it satisfied the more lenient thresholds for fit, giving decent evidence for its generalizability to a new sample (*χ*^*2*^(224) = 1301.34, CFI = .84, RMSEA = .06, SRMR = .07).

Second, we wanted to evaluate whether our new items would help ameliorate some of the shortcomings of the measure identified in Study 2. With respect to the three new and three modified items, we replaced item 50 with item 50*, but other than that, neither the modified items nor the new items made it into our final model. Intellectual modesty item 13 was again one of the worst performing items, and the modified item 13* performed no better. In light of studies 2 and 3, then, we removed item 13 altogether. With these modifications, the model had slightly better fit: *X*^*2*^(231) = 1162.86, CFI = .85, RMSEA = .06, SRMR = .06. The modified scale, which we consider finalized, is displayed in Table 7.

**Table 7:** The final 4-factor solution from study 3.

	OPM	MOD	COR	ENG
27. I think that paying attention to people who disagree with me is a waste of time.	-.70			
34. I feel no shame learning from someone who knows more than me.	.63			
35. If I do not know much about some topic, I don't mind being taught about it, even if I know about other topics.	.60			
45. Even when I have high status, I don't mind learning from others who have lower status.	.62			
50. Only wimps admit that they’ve made mistakes [modified to say “Only wimps admit that they’re confused”].	-.68			
51. I don't take people seriously if they're very different from me.	-.70			
8. Being smarter than other people is not especially important to me.		.60		
10. I would like to be seen explaining ideas that no one else understands.		-.60		
11. I get a lot of pleasure from knowing more than other people.		-.66		
15. I want people to know that I am an unusually intelligent person.		-.68		
32. I like to be the smartest person in the room.		-.77		
37. I find it annoying to be told that I've made an intellectual mistake.			-.73	
38. If someone points out an intellectual mistake that I've made, I tend to get angry.			-.80	
39. I appreciate being corrected when I make a mistake.			.60	
40 When someone corrects a mistake that I've made, I do not feel embarrassed.			.46	
43. When I realize that someone knows more than me, I feel frustrated and humiliated.			-.69	
18. I rarely discuss things that I wish I understood better with other people.				-.59
24. I enjoy reading about the ideas of different cultures.				.46
25. I would be very bored by a book about ideas I disagreed with.				-.54
26. I’ve never really enjoyed figuring out why people disagree with me.				-.52
29. I find it boring to discuss things I don't already understand.				-.68
31. A disagreement is like a war.				-.51

Note. OPM = Open-mindedness, MOD = Intellectual Modesty, COR = Corrigibility, and ENG = Engagement. Factor loading estimates are STYDX. N = 1182.

#### Self-informant agreement

On average, the informants reported that they had known the self-report participant for 14.18 years, and 90.7% of informants reported that they knew the self-report participant “quite well” or “about as well as anybody does.” The highest proportion of informants were friends of the self-report participant (44.9%), followed by family members (19.6%) and romantic partners (17.8%). The lowest proportion of informants were colleagues of the self-report participant (10.3%).

To check for agreement between informant-ratings and self-ratings for each of the subscales, we averaged the informants’ responses on each item for self-report participants who had more than one informant, and then checked how highly the self-report scale scores correlated with those of the scales composed by the aggregated informants’ responses.

The modesty scale had the highest level of agreement between self- and informant-reports (*r* = .47), which is perhaps not surprising, given that the Intellectual Modesty scale reflects the extent to which one is preoccupied with how one is perceived by others (e.g. “I want people to know I am an unusually intelligent person” and “I like to be the smartest person in the room”). The Open-mindedness scale had the next highest level of agreement between self and informant reports (*r* = .34), followed by Corrigibility (*r* = .29) and Engagement (*r* = .28). The level of agreement of the IH- Modesty subscale is comparable to that found for other personality traits. According to three meta-analyses, the average agreement for personality traits is somewhere in the range .40 to .60 [30; 32–33].

The other three subscales have lower agreement than what is typically found for personality traits. There are a few reasons one might expect intellectual humility to have lower agreement than other personality traits. First, agreement tends to be higher for traits that are constituted by more observable behaviors, such as extraversion [29]. Though intellectual humility should lead to certain observable behaviors, these behaviors do not sufficiently define intellectual humility, as certain appropriate attitudes and reactions need to present as well. Second, intellectual humility is a desirable quality, and agreement tends to be lower for more evaluative personality traits [29]. Third, recall that intellectual humility seems to involve a paradox of self-attribution, which suggests that perhaps the informants may be more accurate than the participants in rating the participants’ intellectual humility.

One issue with evaluative characteristics such as intellectual humility is that responses on a self-report measure may be inflated by self-serving biases or “faking good,” obscuring people’s true levels of the trait. One way to evaluate whether this is occurring with our measure is to compare the mean levels of self and informant responses. If people consistently tend to rate themselves as more intellectually humble than their informants do, we might suspect that self-reports are inflated by self-serving biases. In this study, comparison of the mean levels of self- and informant-reports suggest that social desirability bias may indeed play a role in self-reports of intellectual humility, but if it does, it impacts Open-mindedness and Engagement more than the other sub-scales (see Table 8).

**Table 8:** Comparison of informant- and self-ratings of the four IH subscales.

	Informant-ratings *M* (SD)	Self-ratings *M* (SD)	Test of difference
Open-mindedness	5.44 (1.10)	5.78 (0.96)	*t*(88) = 2.63, *p* = .01
Modesty	4.07 (1.31)	4.12 (1.22)	*t*(88) = 0.36, *p* = .72
Corrigibility	4.83 (1.13)	4.96 (1.13)	*t*(87) = 0.87, *p* = .39
Engagement	4.83 (1.01)	5.10 (0.94)	*t*(88) = 2.13, *p* = .04

Note. Means of each subscale are listed with standard deviation in parentheses. A paired samples t-test was also conducted to determine whether there were significant differences between self- and informant-reports of intellectual humility.

### Discussion

Although we had a low response rate from informants, we were able to solicit enough informant reports to perform an initial test of the congruence between self-perceived intellectual humility and other-perceived intellectual humility. The results of this test suggest that our measure is indeed tapping distinct dimensions of a socially recognized construct, at least for informants who have known the main participant for a long time. That said, the statistical relations between self-report and informant-report measures were not as robust as those between self-report measures in this study and those in the previous studies. This may be because people have better or different epistemic access to themselves than the informants have to them. Alternatively, it may be that informants interpret the scale items differently than the main participants, or that they impose looser or different constraints related to consistency than the main participants.

## External validity: Study 4: Test of convergent, divergent, and discriminant validity

Our four-dimensional measure of intellectual humility showed good structural validity across three large samples. Its validity was further supported by convergence across two assessment methods (informant- and self-report). We next wanted to establish external validity by determining the extent to which it displayed convergent, divergent, and discriminant validity. We therefore conducted another study in which a fresh batch of participants responded not only to our measure but also to measures of related, adjacent, and opposed constructs.

### Methods

#### Participants and procedure

Participants (*N* = 980; *M*_age_ = 34.63, *SD*_age_ = 11.22, 454 female) were recruited and compensated using Amazon.com’s Mechanical Turk platform, allowing us to make a direct comparison with the samples from Study 2 and Study 3. Ages ranged from 18–75, and the median education completed was an Associates degree; 47.9% had a Bachelors or higher level of education. 75.5% of participants were White/Caucasian, 8.5% were African-American or Black, 7.9% were Asian, 5.2% were Hispanic, 0.1% were Pacific Islander, .7% were Native American, and 1.1% were Multiracial.

#### Measures

First, we measured over-claiming with the OCQ test [34–35]. In this measure, participants indicate whether they are familiar with a long list of famous people, places, and concepts. However, some items in the list are decoys: they don’t exist. Participants over-claim to the extent that they say they are familiar with these decoys. Second, we measured desirable responding using the BIDR test, which has two facets: self-deceptive enhancement and impression management [36]. Third, we measured trait narcissism using the 13-item scale of grandiose narcissism [37], aggregated into a total score. Fourth, we measured self-esteem using the Rosenberg self-esteem scale [38]. Fifth, we measured grit using the Short Grit Scale [39]. Finally, we measured all dimensions of the Big Six personality model using the 24-item QB6 [40].

Before computing the correlations between scales, we recorded all our expectations for how scales should be related to one another. First, we predicted that Over-claiming Bias would be negatively related to the Open-mindedness subscale. Recall that the negative pole of the Open-mindedness facet is Intellectual Arrogance: it reflects a tendency to be unjustifiably confident about the scope of one’s knowledge. In this regard, over-claiming is a direct behavioral manifestation of the negative pole of Open-mindedness. Someone who is open-minded should be less prone to over-claiming knowledge than someone who is intellectually arrogant.

Trait narcissism is characterized by both grandiosity and attention-seeking/exhibitionism [37]. Therefore, we hypothesized that Narcissism would be negatively related to Open-mindedness, since the negative pole of the IH-Open-mindedness scale involves a sense of grandiosity toward one’s ideas, as well as negatively related to IH-Modesty, since IH-Modesty involves a certain level of exhibitionism and desire for admiration for one’s intellectual qualities.

We hypothesized that Grit—defined by Duckworth et al. [41]—as perseverance and passion for long-term goals) would be positively related to the Engagement subscale, since like Grit, the Engagement subscale involves tenacity applied to understanding things one does not already understand and ideas different from one’s own.

With respect to personality traits, we expected that Open-mindedness would have a positive correlation with the QB6-Honesty scale, since the Honesty dimension of personality involves a prioritization of moral standards and the welfare of others over the advancement of one’s own status, and the IH-Open-mindedness scale captures a lack of regard for the status that may come with intellectual accomplishment. Second, we hypothesized that IH-Corrigibility would have a positive correlation with the QB6-Resilience factor since it primarily taps one’s emotional responses to being intellectually criticized or making intellectual mistakes. People higher in Resilience should be more likely to take potential challenges to their intellect in stride. Third, we hypothesized that IH-Engagement would have a positive correlation with QB6-Originality/Intellect, since people who are higher on Originality/Intellect should be more motivated to understand ideas different from their own.

We included the Balanced Inventory of Desirable Responding (BIDR) scales to see to what extent socially desirable responding affected responses to the IH subscales. We hoped that the correlation between the intellectual humility (IH) scales and BIDR scales would be relatively small, at least smaller than the correlations between the intellectual humility scales and the constructs we expected to be related for substantive reasons.

We did not have predictions for the Over-claiming accuracy score, or the Rosenberg self-esteem scale [38]. We included the self-esteem scale in our study mainly to ensure that we were tapping into intellectual humility rather than some form of diffidence, since semantically, humility is very close in meaning to qualities denoting a lack of healthy self-esteem, such as meekness and subservience [10]. Thus, with respect to the self-esteem scale, we mainly wanted to make sure that we did not find a negative correlation between the four facets of intellectual humility and self-esteem.

Beyond the relationships discussed above, we did not have any predictions about what other relationships we would find between the IH scales and the scales we included in our study.

### Analyses

For most of the scales, responses were averaged as long as the participant had answered 70% or more of the questions on that scale, following missing data procedures recommended by Graham [42]. The percentage of people with incomplete data (at least one or more scores at the scale-level missing) was 2.6%.

We used pairwise deletion when computing correlations between scales. Newman [43] suggests pairwise deletion does not bias estimates or undermine power, as long as the percentage of respondents with any missing data does not exceed 10%.

### Results

We first checked the fit of the four-factor model of intellectual humility in a SEM framework. Using ML estimation with robust standard errors (*N* = 980) we found the four-factor model had similar fit to that found previously, *χ*^2^(224) = 1047.09, CFI = .87, RMSEA = .06, SRMR = .06. Missing data for the latent variable model was handled using the Mplus default method, Full Information Maximum Likelihood.

All our predictions were confirmed in that the constructs we expected to have a relationship had a significant relationship in the direction we predicted (see Table 9). With respect to personality traits, all our predictions were also accurate: IH-Open-mindedness had a positive correlation with QB6-Honesty (*r*(977) = .35, IH-Corrigibility had a positive correlation with QB6-Resilience (*r*(977) = .41), and IH-Engagement had a positive correlation with QB6-Intellect, *r*(975) = .42.

**Table 9:** Correlations between IH scales and related constructs.

	OCQ-Bias	Narciss-ism	Grit	OCQ-Accuracy	BIDR-SDE	Self-Esteem	BIDR-IM
Open-mindedness	–.16**	–.09*	.20**	.27**	.33**	.28**	.37*
Modesty	–.09*	–.40**	.16**	–.01	.09*	.12**	.19**
Corrigibility	–.04	.06	.36**	–.05	.40**	.39**	.34**
Engagement	.01	.04	.25**	.17**	.30**	.31**	.27**

Note. In bold are correlations that we predicted a priori. Correlations calculated with *N* between 956 and 979.

**Correlations is significant at the .001 level (2-tailed).

*Correlation is significant at the .01 level (2-tailed).

Though our results were consistent with our predictions, a more stringent criterion for construct validity is the relative relationship between constructs we expected to be related and those we did not expect to be related. For example, the BIDR scales had correlations with the Open-mindedness, Corrigibility, and Engagement scales that were similar in magnitude to correlations between constructs we predicted should be related for substantive reasons. In addition, our scales were related to more personality dimensions than we expected (see Table 10).

**Table 10:** Correlations between IH scales and Big Six scales.

	QB6 Honesty	QB6 Resil.	QB6 Intellect	QB6 Extrav.	QB6 Agree.	QB6 Consci.
Open-mindedness	**.35**	.24	.34	.31	.37	.18
Modesty	.29	.11	–.20	.11	.21	.11
Corrigibility	.23	**.41**	.25	.36	.51	.25
Engagement	.20	.27	**.42**	.33	.36	.18

Note. In bold are correlations that we predicted a priori. All correlations are significant at the .001 level (2-tailed). Correlations calculated with *N* between 977 and 979.

Though not quite what we predicted, our results suggest some interesting testable hypotheses about the origins of intellectual humility and the consequences of being intellectually humble. The pattern of correlations we found is consistent with the idea that to be intellectually humble, one must have a certain level of healthy psychological adjustment and self-esteem (Corrigibility, Engagement, and Open-mindedness had the strongest relationships to Self-Esteem, ranging from *r* = .28 to *r* = .39). A general factor of adjustment is thought to underlie responses to inventories measuring desirable personality traits as well, so this is consistent with that idea. In addition, we found that IH-Open-mindedness was strongly related to OCQ-Accuracy scores, suggesting there may be a relationship between IH-Open-mindedness and some form of crystallized intelligence. Though not what we predicted, beneficial intellectual outcomes such as increased familiarity with a number of topics feasibly could be a consequence of having intellectual humility, as intellectual humility should increase one’s ability to reform one’s beliefs appropriately when encountering new information.

The generally high correlations between intellectual humility and personality dimensions raise a question about whether this scale predicts relevant behaviors above and beyond what already developed models of personality can predict. Is intellectual humility just a combination of the personality traits Honesty/Propriety, Openness, and Agreeableness, and thus is a new scale for it unnecessary? To test this, we performed a hierarchical regression where we tested how much the IH-Open-mindedness subscale predicted Over-claiming Bias above and beyond what the six QB6 scales could predict. Over-claiming is a direct behavioral manifestation of intellectual humility, and thus served as our outcome criterion for whether the Open-mindedness scale might be a useful measure of individual differences that can supplement already-existing personality measures. We found that the QB6 personality scales combined explained a significant proportion of variance in Over-claiming Bias, *F*(6, 951) = 8.75, *p* < .001, *R*^*2*^ = .05, but the only personality traits in this model with significant coefficients were QB6-Honesty (*β* = –0.18), QB6-Intellect (*β* = 0.12), and QB6-Agreeableness (*β* = 0.09). In the second step, IH-Open-mindedness explained a significant amount of variance in Over-claiming Bias above and beyond what the QB6 traits accounted for, *F*_*change*_(1, 950) = 33.26, *p* < .001, *R*^*2*^_*chang*e_ = .03. In the full regression model with the QB6 traits and Open-mindedness, Open-mindedness was the strongest predictor of Over-claiming Bias (*β* = –0.21, *t*(950) = –5.77, *p* < .001).

The fact that the IH-Open-mindedness subscale uniquely predicts more than half the amount of variance that all six personality traits combined predict in Over-claiming Bias suggests that this measure scale is tapping into a meaningful individual difference that is distinct from personality as measured by the QB6.

### Discussion

We found that our measure of intellectual humility was negatively related to dispositions that are intuitively opposed to intellectual humility, and positively related to adjacent constructs, while not merely redundant with them. Second, the four-factor structure of intellectual humility replicated yet another time in a new sample, suggesting once again that we are tapping a real aspect of intellectual personality or character. Third, our four subscales predict meaningfully distinct outcomes, giving credence to their divergent validity. IH-Corrigibility may have the most basis in personality: of all the subscales, it shows the highest relation to multiple personality dimensions. Perhaps most telling is the fact that IH-Open-mindedness most strongly predicts behavioral outcomes such as Over-claiming one’s knowledge, while IH-Modesty is most strongly related to trait narcissism. Of all the subscales, IH-Open-mindedness and IH-Engagement may be most relevant for predicting crystallized knowledge or other forms of intelligence, as indicated by their relatively high correlations with OCQ-Accuracy. We should also note that one of the other extant scales of intellectual humility [11] has been tested as a predictor of OCQ-Bias by Deffler, Leary, & Hoyle [44], with a null result. Of course, this does not mean that the Leary et al. [11] scale is incapable of predicting over-claiming, but the track record of our scale is demonstrably better. This may be because the multi-factorial nature of our scale enables it to capture the construct more fully, adding predictive power beyond that of the Leary et al. scale

## External validity: Study 5: German-language replication

The first successful replications of the Big Five model outside of English were in German [45]. This suggests that our intellectual humility scale is especially likely to replicate in German, though we hope eventually to study other languages. In this study, we translated all items and tested the extent to which Germanophone participants’ responses to them had the same structure as the responses of our earlier Anglophone participants.

### Methods

#### Participants and procedure

Because Mechanical Turk is not a viable recruitment venue for German-speaking participants, we used the participant recruitment system of the University of Zurich, which allows researchers to approach students and staff of all faculties to recruit a sample of convenience. In total, 579 participants provided valid answers. The average age of participants was 34.5; 38.7% of the sample was male. The distribution across faculties was as follows: philosophy 4.5%, psychology 12.1%, other humanities discipline 24.4%, social sciences 13.0%, sciences 18.1%, law 9.5%, medicine 12.8%, other 5.7%. 39.0% of the sample had a master’s degree, 31.1% had a PhD. Thus, this sample is very highly-educated compared to our previous samples, reflecting the fact that many of our recruits were graduate students, postdocs, or faculty. In future work, it would be worthwhile to replicate this study by sampling a more educationally diverse population. We used a random sub-sample of 279 participants for exploratory factor analysis and the remaining 300 participants for confirmatory factor analysis. Thus, this study replicates in German both study 1 and study 2 above.

#### Measure

The goal of the German-language study was to precisely replicate the procedure that we used in the English study. This approach should allow better identification of cultural differences regarding the understanding of intellectual humility, such as differences in the composition of the factors. Therefore, we refrained from simply translating the final items of the four-factor English scale. Instead, all 52 items of the original English scale were translated by one author, and the translations were checked independently by two experts who were not involved in the study (one social psychologist and one English teacher in a technical university). A complete list of translated items is available in S2 Table. When comparing the German with the English result, we looked for configural invariance; that is, with both versions having same factor structure.

### Results

We used the same methodology for determining the factor structure as outlined in studies 1 and 2. In the exploratory factor analysis, parallel analysis suggests that the number of factors was 10 and the number of components was 7. Given this situation and to enable a comparison with the result of the English study, we used the 8-factor solution (χ^2^(938) = 1278.17, *p <* 0.001), as this was also more interpretable. The result is outlined and compared with the result of the English EFA in Table 11; the item numbers match with the translations. We find that three factors show a complete match (Intellectual Machiavellianism, Intellectual Kleptomania, Corrigibility) and two factors show an item-overlap of 50% and more (Engagement and Curiosity).

**Table 11:** Comparing the German with the English exploratory factor analysis.

Name of English Factor	Item # ENG	Item # GER	Name of German Factor
Intellectual Machiavellianism	**1, 2, 3, 4**	**1, 2, 3, 4**	Lobhudelei
Intellectual Kleptomania	**5, 6, 7**	**5, 6, 7**	Ideenklau
Corrigibility	**37, 38, 39, 40,** 43	**37, 38, 39, 40**	Verbesserungsfähigkeit
Engagement	18, 24, 25, 26, 29, 31		Offenheit für Neues
Curiosity	**19, 20, 21, 22**	**19, 20, 21, 22,** 23, 26, 36	Neugier
Open-mindedness	17, 23, **27, 28, 33**, 34, 35, 36, 39, 41, 45, 50, **51**, 52	25, **27, 28,** 31, **33, 51**	Aufgeschlossenheit
Intellectual Modesty	**8,** 9, **10, 11, 12, 13, 14, 15,** 16, **32**	**8**, **10, 11**, **12**, **13, 14, 15, 32,** 41, 42, 44, 46	Selbstbescheidung
Intellectual Uniqueness	42, 44, 46		[semantically inconsistent]
Excluded items	30, 47, **48, 49**	9, 14, 16, 18, 20, 24, 28, 29, 34, **48, 49**	

Note. Items present in corresponding factors are printed in bold.

For this reason, we used the four-factor solution derived from the English language CFA (i.e., Study 2). Applying this solution in the German sample produced adequate fit, *χ*^*2*^(224) = 359.89, *p* < 0.001, CFI = 0.88, RMSEA = 0.05, SRMR = 0.06. The model was fitted using robust standard errors, Satorra-Bentler’s scaled *χ*^2^ and full-information maximum likelihood to handle missingness.

The resulting fit indices are comparable to the English language version. This suggests that the factor structure of the German version of the scale is similar to the factor structure of the original English version. The factor loadings for most of the factors also look similar in German as they did in English, suggesting that these items relate to their factors similarly across German and English. The Open-mindedness/Aufgescholossenheit factor performs less well, however; the factor loadings for this factor are generally lower in the German solution compared to the English solution, and the Open-mindedness/Aufgescholossenheit item 34 is the only item that did not load on its factor. Table 12 illustrates the German version of the four-factor solution.

**Table 12:** The 4-factor solution (German version).

	OPM	MOD	COR	ENG
27. Ich glaube, sich mit Leuten abzugeben, die anderer Meinung sind als ich, ist Zeitverschwendung.	-.63			
34. Es beschämt mich nicht, etwas von einer Person zu lernen, die mehr weiss als ich.	.07			
35. Wenn ich über ein Thema wenig weiss, macht es mir nichts aus, darüber belehrt zu werden, auch wenn ich über andere Themen viel Bescheid weiss.	.47			
45. Selbst wenn mein Stellenwert hoch ist, habe ich kein Problem etwas von jemandem zu lernen, der einen tieferen hat.	.40			
50. Nur Schwächlinge geben zu, dass sie einen Fehler gemacht haben.	-.48			
51. Ich nehme Leute nicht ernst, die sich sehr von mir unterscheiden.	-.54			
8. Klüger als andere zu sein, ist nicht wirklich wichtig für mich.		.72		
10. Ich würde gerne als einer angesehen, der Dinge erklären kann, die sonst niemand versteht.		-.60		
11. Es würde mir viel Befriedigung verschaffen, mehr zu wissen als andere		-.70		
13. Ich möchte nicht, dass andere mich behandeln, als ob ich ihnen intellektuell überlegen wäre.		.50		
15. Ich will, dass die andern wissen, dass ich eine aussergewöhnlich intelligente Person bin.		-.67		
32. Ich bin gerne die klügste Person im Raum.		-.67		
37. Es stört mich wenn andere mir sagen, ich hätte einen Denkfehler gemacht.			-.78	
38. Wenn jemand auf einen Denkfehler hinweist, den ich gemacht habe, kann mich das verärgern.			-.73	
39. Ich schätze es, korrigiert zu werden, wenn ich einen Fehler mache.			.52	
40. Wenn jemand einen Fehler von mir korrigiert, bringt mich das nicht in Verlegenheit.			.44	
43. Wenn ich realisiere, dass jemand mehr als ich weiss, fühle ich mich frustriert und gedemütigt.			-.55	
18. Ich diskutiere mit anderen Leuten selten über Dinge, die ich gerne besser verstehen würde.				-.46
24. Ich lese gerne über Ideen anderer Kulturen.				.33
25. Es würde mich langweilen ein Buch über Ideen zu lesen, mit denen ich nicht einverstanden bin.				-.43
26. Es hat mich bislang nie wirklich interessiert herauszufinden, warum Leute anderer Meinung sind als ich.				-.53
29. Es langweilt mich über Dinge zu diskutieren, die ich nicht bereits verstehe.				-.49
31. Eine Meinungsverschiedenheit ist wie ein Krieg.				-.45

Note. OPM = Open-mindedness/Aufgescholossenheit, MOD = Intellectual Modesty/ Aufgescholossenheit, COR = Corrigibility/Aufgescholossenheit, and ENG = Engagement/ Offenheit für Neues. Factor loading estimates are STYDX.

### Discussion

It would be premature to say that the conceptions of intellectual humility in German and English are identical. A single study with a modest sample size of highly-educated Germanophone Swiss participants is not sufficient to draw any such conclusions. Nevertheless, the broad outlines of intellectual humility appear similar in English and German. Further research may help to determine the extent to which the similarities and differences can be attributed to measurement error, lack of item clarity, differences in basic psychological dispositions or conceptions of personality, and so on. Of particular value will be multi-modal studies that go beyond self-report, as in study 3.

## Conclusions

In this paper, we presented evidence from five studies on the development and validation of a scale of intellectual humility. This scale captures cognitive, affective, behavioral, and motivational components of the construct that have been identified by various philosophers in their conceptual analyses of intellectual humility. Using these analyses to inform a broad item pool, four core dimensions emerged from self-evaluations of U.S. college students, and this structure replicated in two large non-student U.S. samples. These four core dimensions included: Open-mindedness (as opposed to Intellectual Arrogance), Engagement (as opposed to Boredom), Intellectual Modesty (as opposed to Intellectual Vanity), and Corrigibility (as opposed to Intellectual Fragility).

The validity of these subscales was supported by convergence between self-reports and informant-reports in Study 3, and these subscales’ abilities to predict relevant outcomes in Study 4. These results indicate that future research should look more carefully at the relationships between intellectual humility, domain knowledge, and various aspects of intelligence. Intellectual humility adds predictive power beyond that of the Big Six model of personality, suggesting it taps an important individual difference that is not redundant with already-established personality models.

Finally, a similar factor structure emerges when the items of our scale are translated into German and responded to by Germanophone participants, giving initial evidence for the relevance of intellectual humility in non-U.S. populations. However, work in countries outside of Western and central Europe is needed to better evaluate the cross-cultural relevance of intellectual humility as measured in this study. As we mentioned above, the first successful replications of the Big Five model outside of English were in German. The next language in which replication was successful was Dutch, so a natural next step would be to reproduce this research in the Netherlands. Naturally, it would also be very interesting and worthwhile to reproduce this research outside of North America and Western Europe—for instance in China and Japan, not to mention sub-Saharan Africa.

Another area for future research on intellectual humility is predicting important consequences and behaviors based on our scale of intellectual humility. One naturally expects that intellectually humble people would promote the epistemic flourishing of their collaborators (perhaps even at an epistemic cost to themselves) in problem-solving social contexts, whereas intellectually arrogant people would promote their own epistemic flourishing (perhaps even at an epistemic cost to their collaborators). In addition, it seems plausible that intellectually humble people would be better able to engage in public discourse about contentious and controversial topics, such as the health consequences of vaccination and reparations for atrocities committed by previous generations (e.g., American chattel slavery and Jim Crow). More generally, it would be worthwhile to establish the real-world consequences of being high or low in the four factors of intellectual humility identified in this paper.

Finally, it would be worthwhile to conduct comparative studies using the scale developed hear alongside those developed by Leary et al. [11], Krumrei-Mancuso & Rouse [12], and McElroy et al. [13]. We have already noted above some reasons to prefer our scale, but a direct comparison would be the simplest way to make such a decision.

We hope to have laid a strong foundation for such future research.

## Supporting information

S1 Table Initial item pool in English. This is the initial item pool in English, classified based on the exploratory factor analysis in study 1. Reverse-keyed items are indicated by (–).
(DOCX)

S2 Table Initial item pool in German. This is the initial item pool in German, classified based on the exploratory factor analysis in study 5. The numbering is identical to the English original. Reverse-keyed items are indicated by (–).
(DOCX)
