# Supplementary material for: Development and validation of a multi-dimensional measure of intellectual humility
Source: PLoS One. 2017 Aug 16;12(8):e0182950. doi: 10.1371/journal.pone.0182950 (PMC5559088; doi:10.1371/journal.pone.0182950)
Supplement: S1 Table — This is the initial item pool in English, classified based on the exploratory factor analysis in study 1. Reverse-keyed items are indicated by (–). (DOCX) [file pone.0182950.s001.docx]

**S1 Table.**

| Open-Mindedness | |
| --- | --- |
| 17 | Whenever I don’t understand something, I want to ask another person for guidance. |
| 23 | When someone disagrees with me, I try to find out why. |
| 27 | (–) I think that paying attention to people who disagree with me is a waste of time. |
| 28 | I like talking to people with different viewpoints than mine. (This item also had a loading above .3 on Boredom.) |
| 33 | (–) When a person disagrees with me, I usually assume there is something wrong with that person. |
| 34 | I feel no shame learning from someone who knows more than me. |
| 35 | If I do not know much about some topic, I don’t mind being taught about it, even if I know a lot about other topics. [modified to say “Even though I’m well-versed in some things, I don’t mind being taught about other things.”] |
| 36 | Teachers can learn a lot from their students. |
| 39 | I appreciate being corrected when I make a mistake. |
| 41 | I would never brag about how much I know. |
| 45 | Even when I have high status, I don’t mind learning from others who have lower status. |
| 50 | (–) Only wimps admit that they’ve made mistakes [modified to say “Only wimps admit that they’re confused”]. |
| 51 | (–) I don’t take people seriously if they’re very different from me. |
| 52 | (–) People from other countries have weird ideas. |
| Intellectual Modesty | |
| 8 | Being smarter than other people is not especially important to me. |
| 9 | (–) I would like to have more access to information than everyone else. |
| 10 | (–) I would like to be seen explaining ideas that no one else understands. |
| 11 | (–) I would get a lot of pleasure from knowing more than other people. |
| 12 | I don’t feel that I know or understand more than most other people. |
| 13 | I wouldn’t want people to treat me as though I were intellectually superior to them. [modified to say “I wouldn’t want people to treat me as though I were a genius.”] |
| 14 | (–) I think I am entitled to more intellectual respect than the average person is. |
| 15 | (–) I want people to know that I am an unusually intelligent person. |
| 16 | (–) I can solve difficult puzzles without needing intellectual support from anyone else. |
| 32 | (–) I like to be the smartest person in the room. |
| Corrigibility | |
| 37 | (–) I find it annoying to be told that I’ve made an intellectual mistake. |
| 38 | (–) If someone points out an intellectual mistake that I’ve made, I tend to get angry. |
| 39 | I appreciate being corrected when I make a mistake. |
| 40 | When someone corrects a mistake that I’ve made, I do not feel embarrassed. |
| 43 | (–) When I realize that someone knows more than me, I feel frustrated and humiliated. |
| Engagement | |
| 18 | (–) I rarely discuss things that I wish I understood better with other people. |
| 24 | I enjoy reading about the ideas of different cultures. |
| 25 | (–) I would be very bored by a book about ideas I disagreed with. |
| 26 | (–) I’ve never really enjoyed figuring out why people disagree with me. |
| 29 | (–) I find it boring to discuss things I don’t already understand. |
| 31 | (–) A disagreement is like a war. |
| Curiosity | |
| 19 | I try to learn as much as I can. |
| 20 | I often push myself very hard when trying to master a new idea. |
| 21 | (–) Often when I’m in the process of learning something, I end up quitting without having really mastered it. |
| 22 | (–) I learn only the minimum amount needed to get by. |
| Uniqueness | |
| 42 | I don’t feel special when I realize that I know a lot. |
| 44 | If I know a lot about some topic, I don’t feel special about it. |
| 46 | I don’t feel special when I realize that I know more than other people. |
| Intellectual Machiavellianism | |
| 1 | If I want someone I dislike to tell me what I want to know, I will act very nicely toward that person in order to get the information. |
| 2 | (–) I wouldn’t use flattery to get information from someone, even if I thought it would succeed. |
| 3 | If I want information from someone, I will laugh at that person’s worst jokes. |
| 4 | (–) I wouldn’t pretend to like someone just to get that person to tell me what I want to know. |
| Intellectual Kleptomania | |
| 5 | If I knew I could never get caught, I would be willing to steal someone else’s ideas. |
| 6 | I would be tempted to steal someone else’s ideas if I didn’t have the time or interest to come up with my own ideas. |
| 7 | I’d be tempted to copy someone else’s work, if I were sure I could get away with it. |
| Excluded Items (not loading above .3 on any factor or cross loading) | |
| 28 | I would rather be convincing but wrong than unconvincing but right. |
| 30 | I like talking to people with different viewpoints than mine. |
| 47 | I have a good understanding of what I know and what I don’t know. |
| 48 | Being prejudiced against someone in a discussion is not a big problem. |
| 49 | When arguing with someone, tend to exaggerate how much I know. |
