# Supplementary material for: Development and validation of a multi-dimensional measure of intellectual humility
Source: PLoS One. 2017 Aug 16;12(8):e0182950. doi: 10.1371/journal.pone.0182950 (PMC5559088; doi:10.1371/journal.pone.0182950)
Supplement: S2 Table — This is the initial item pool in German, classified based on the exploratory factor analysis in study 5. The numbering is identical to the English original. Reverse-keyed items are indicated by (–). (DOCX) [file pone.0182950.s002.docx]

**S2 Table**

| Aufgeschlossenheit | | |
| --- | --- | --- |
| 25 | (–) Es würde mich langweilen ein Buch über Ideen zu lesen, mit denen ich nicht einverstanden bin. | |
| 27 | (–) Ich glaube, sich mit Leuten abzugeben, die anderer Meinung sind als ich, ist Zeitverschwendung. | |
| 28 | Ich unterhalte mich gerne mit Leuten, die andere Ansichten haben als ich. | |
| 31 | (–) Eine Meinungsverschiedenheit ist wie ein Krieg. | |
| 33 | (–) Wenn jemand mir widerspricht, nehme ich normalerweise an, dass mit dieser Person irgendwas nicht stimmt. | |
| 51 | (–) Ich nehme Leute nicht ernst, die sich sehr von mir unterscheiden. | |
| Neugier | | |
| 19 | Ich versuche so viel wie möglich zu lernen. | |
| 20 | Ich strenge mich oft sehr an, wenn ich eine neue Idee verstehen will. | |
| 21 | (–) Wenn ich daran bin, etwas zu lernen, ist es häufig so, dass ich aufgebe, bevor ich es wirklich verstanden habe. | |
| 22 | (–) Ich lerne nur das Minimum, um gerade noch durchzukommen. | |
| 23 | Wenn jemand anderer Meinung ist als ich, versuche ich herauszufinden, warum dies so ist. | |
| 26 | (–) Es hat mich bislang nie wirklich interessiert herauszufinden, warum Leute anderer Meinung sind als ich. | |
| 36 | Lehrpersonen können von ihren Schülerinnen und Schülern viel lernen. | |
| Selbstbescheidung | | |
| 8 | Klüger als andere zu sein, ist nicht wirklich wichtig für mich. |  |
| 10 | (–) Ich würde gerne als einer angesehen, der Dinge erklären kann, die sonst niemand versteht. |  |
| 11 | (–) Es würde mir viel Befriedigung verschaffen, mehr zu wissen als andere. |  |
| 12 | Ich glaube nicht, dass ich mehr weiss oder verstehe als andere. |  |
| 13 | Ich möchte nicht, dass andere mich behandeln, als ob ich ihnen intellektuell überlegen wäre. |  |
| 14 | (–) Ich glaube, dass ich grösseren intellektuellen Respekt verdiene als durchschnittliche Menschen. |  |
| 15 | (–) Ich will, dass die andern wissen, dass ich eine aussergewöhnlich intelligente Person bin. |  |
| 32 | (–) Ich bin gerne die klügste Person im Raum. |  |
| 41 | Ich würde nie damit prahlen, wieviel ich weiss. |  |
| 42 | Ich fühle mich nicht als jemand Besonderes, wenn ich erkenne, dass ich viel weiss. |  |
| 44 | Wenn ich zu einem bestimmten Thema viel weiss, fühle ich mich deshalb nicht speziell. |  |
| 46 | Klüger als andere zu sein, ist nicht wirklich wichtig für mich. |  |
| Verbesserungsfähigkeit | | |
| 37 | (–) Es stört mich wenn andere mir sagen, ich hätte einen Denkfehler gemacht. | |
| 38 | (–) Wenn jemand auf einen Denkfehler hinweist, den ich gemacht habe, kann mich das verärgern. | |
| 39 | Ich schätze es, korrigiert zu werden, wenn ich einen Fehler mache. | |
| 40 | Wenn jemand einen Fehler von mir korrigiert, bringt mich das nicht in Verlegenheit. | |
| Lobhudelei | | |
| 1 | Wenn ich von jemandem, den ich nicht mag etwas wissen will, dann werde ich mich dieser Person gegenüber sehr freundlich benehmen, um die Information zu erhalten. | |
| 2 | (–) Ich würde niemandem schmeicheln, um zu Informationen zu kommen, selbst wenn ich glaube, dass ich Erfolg haben könnte. | |
| 3 | Wenn ich von jemandem etwas wissen will, werde ich über die faulsten Witze dieser Person lachen. | |
| 4 | (–) Ich würde keiner Person vorspielen sie zu mögen, nur um von ihr zu erfahren, was ich wissen will. | |
| Ideenlklau | | |
| 5 | Wenn ich sicher wäre, dass ich nicht erwischt würde, würde ich die Ideen anderer stehlen. | |
| 6 | Ich wäre versucht, die Idee eines anderen zu stehlen, wenn ich keine Zeit oder kein Interesse hätte, eigene Ideen zu entwickeln. | |
| 7 | Ich wäre versucht, die Arbeit eines anderen zu kopieren, wenn ich sicher wäre, dass ich damit durchkommen würde. | |
| Items not loading above .3 on any factor (or cross-loading) | | |
| 9 | (–) Ich hätte gerne mehr Zugang zu Informationen als alle anderen. | |
| 16 | (–) Ich kann schwierige Rätsel ohne fremde Hilfe lösen. | |
| 17 | Immer wenn ich etwas nicht verstehe, möchte ich eine andere Person um Rat fragen. | |
| 18 | Ich diskutiere mit anderen Leuten selten über Dinge, die ich gerne besser verstehen würde. | |
| 24 | Ich lese gerne über Ideen anderer Kulturen. | |
| 29 | (–) Es langweilt mich über Dinge zu diskutieren, die ich nicht bereits verstehe. | |
| 30 | Ich wirke lieber überzeugend und bin im Unrecht, als nicht überzeugend zu wirken und Recht zu haben. | |
| 34 | Es beschämt mich nicht, etwas von einer Person zu lernen, die mehr weiss als ich. | |
| 35 | Wenn ich über ein Thema wenig weiss, macht es mir nichts aus, darüber belehrt zu werden, auch wenn ich über andere Themen viel Bescheid weiss. | |
| 43 | (–) Wenn ich realisiere, dass jemand mehr als ich weiss, fühle ich mich frustriert und gedemütigt. | |
| 45 | Selbst wenn mein Stellenwert hoch ist, habe ich kein Problem etwas von jemandem zu lernen, der einen tieferen hat. | |
| 47 | Ich kann gut einschätzen, was ich weiss und was ich nicht weiss. | |
| 48 | (–) Vorurteile gegenüber einem andern ist in einer Diskussion kein grosses Problem. | |
| 49 | Wenn ich mit jemandem diskutiere, neige ich dazu, meine Sachkompetenz zu übertreiben. | |
| 50 | (–) Nur Schwächlinge geben zu, dass sie einen Fehler gemacht haben. | |
| 52 | (–) Leute aus anderen Ländern haben seltsame Ideen. | |
